# Factors associated with physical growth of children during the first two years of life in rural and urban areas of Vietnam

**DOI:** 10.1186/1471-2431-13-149

**Published:** 2013-09-25

**Authors:** Huong Thu Nguyen, Bo Eriksson, Max Petzold, Göran Bondjers, Toan Khanh Tran, Liem Thanh Nguyen, Henry Ascher

**Affiliations:** 1 Research Institute for Child Health, National Hospital of Pediatrics, 18/879 La Thanh Road, Hanoi, Dong Da district, Vietnam; 2 Nordic School of Public Health, PO Box 12133, Gothenburg, SE-402 42, Sweden; 3 Family Medicine Department, Hanoi Medical University, No.1 Ton That Tung Street, Hanoi, Vietnam; 4 Sahlgrenska Academy, University of Gothenburg, PO Box 440, Gothenburg, SE-405 30, Sweden

**Keywords:** Growth of children, Antenatal care, Breastfeeding, Reported illness, Rural and urban area

## Abstract

**Background:**

Differences between urban and rural settings can be seen as a very important example of gaps between groups in a population. The aim of this paper is to compare an urban and a rural area regarding child growth during the first two years of life as related to mother’s use of antenatal care (ANC), breastfeeding and reported symptoms of illness.

**Methods:**

The studies were conducted in two Health and Demographic Surveillance Sites, one rural and one urban in Hanoi, Vietnam.

**Results:**

We found that children in the urban area grow faster than those in the rural area. There were statistical associations between growth and the education of the mother as well as household resources. There were positive correlations between the number of ANC visits and child growth. We also saw a positive association between growth and early initiation (first hour of life) of breastfeeding but the reported duration of exclusive breastfeeding was not statistically significantly related to growth. Reporting symptoms of illness was negatively correlated to growth, i.e. morbidity is hampering growth.

**Conclusions:**

All predictors of growth discussed in this article, ANC, breastfeeding and illness, are associated with social and economic conditions. To improve and maintain good conditions for child growth it is important to strengthen education of mothers and household resources particularly in the rural areas. Globalization and urbanization means obvious risks for increasing gaps not least between urban and rural areas. Improvement of the quality of programs for antenatal care, breastfeeding and integrated management of childhood illness are also needed in Vietnam.

## Background

Birth weight and child growth are important predictors for the future health of a person and for the public health of a population. Abnormal growth in utero and during infancy can have immediate negative effects but may also lead to adverse health effects later in life e.g. as stated by the Barker hypothesis [1]. Suboptimal growth during fetal life and infancy can influence weight gain in childhood and increase risk of hypertension, coronary heart disease and type II diabetes later in life [2,3]. These diseases are today major public health challenges, established in high income countries and emerging in many low and middle income countries. Epidemiological transition from communicable to non-communicable diseases, or to a combination of both, poses a major public health problem involving the whole or large groups of a population [4,5]. Recent studies in Vietnam point in the same direction [5]. The growth of children is a complex process that depends on many interacting factors including both genes and environment. Particularly important are the prenatal and postnatal nutritional status of the mother as well as infant factors such as birth weight, diet and infections. These factors are in turn and to different degrees determined by socioeconomic, cultural and biologic conditions [6].

**Figure 1:**
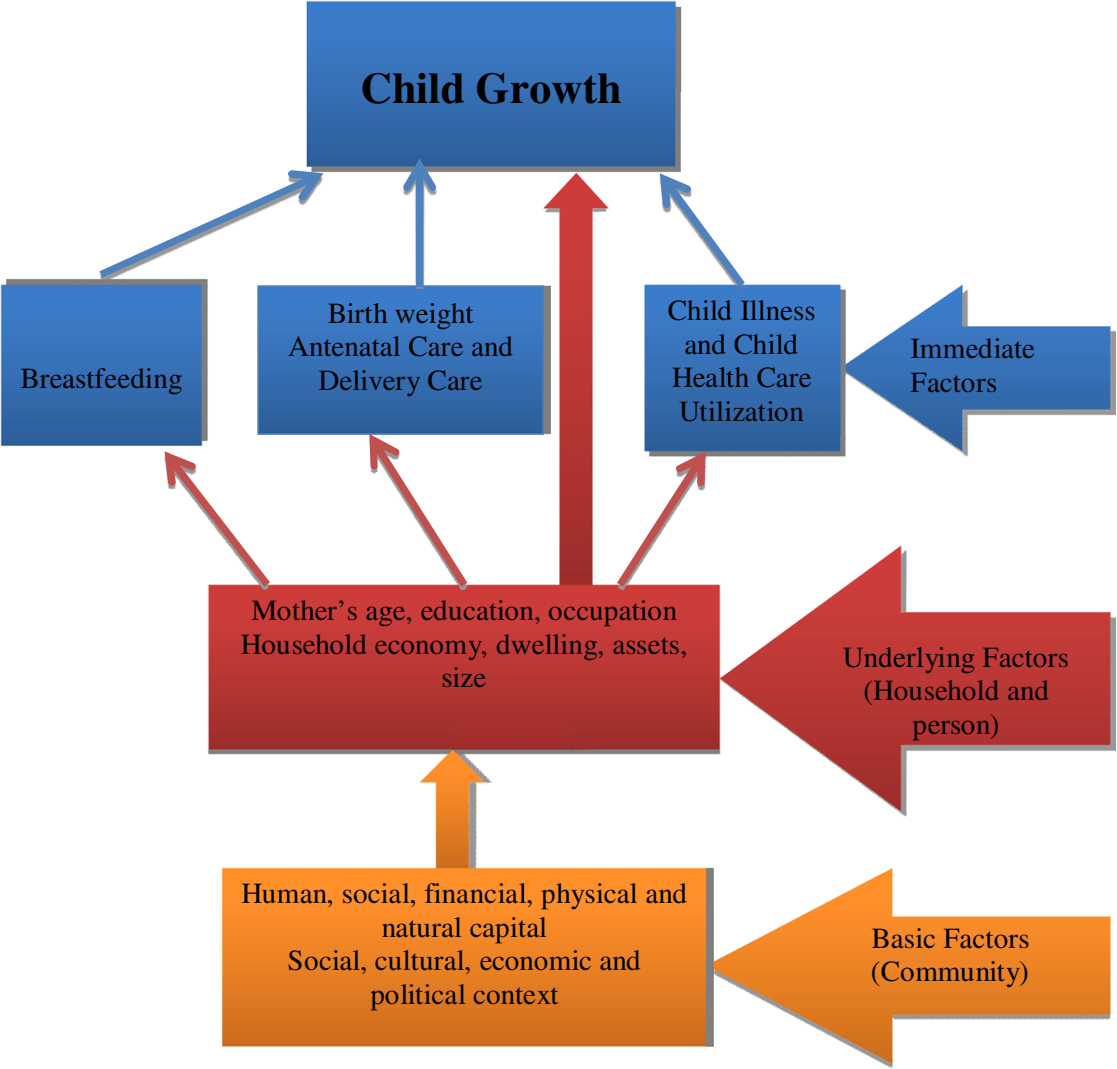
Conceptual framework of this paper.

The conceptual framework for this paper (Figure 1) is based on the one given for malnutrition by Black et al. [7] however modified for the present situation.

The most important *basic factors* possibly indirectly influencing child growth are the general social, cultural, economic and political contexts. These are fundamental for establishing human, social, financial, physical and natural capital, all determinants for living conditions and distinctively different between urban and rural contexts.

*Underlying factors* are primarily characteristics of persons and households. For persons, the traditional demographic factors like the age of the mother and, to some extent, of the father are of interest. Education and occupation of parents [8,9] particularly of the mother, can be expected to be of importance. Children with mothers having higher education have shown better growth (lower stunting, underweight, obesity and overweight) [8]. At the household level, economy, dwelling characteristics, assets and size, numbers of adults and children, are key factors [9,10]. Satisfactory personal and household social and economic resources are needed as underlying factors to create conditions and interest for health promoting choices and behavior.

*Immediate factors* are directly influencing child growth at the individual level. The birth weight of a child is the result of intrauterine growth as well as the nutritional conditions and gestational age at birth. It is also reflecting the mother’s health, nutritional status and behavior during pregnancy including e.g. use of antenatal care and smoking. Birth weight is the starting point for the infant’s further growth. After birth, nutritional practices, primarily breastfeeding, and child illness are likely to influence growth.

This article will study three immediate factors, antenatal care (ANC), breastfeeding and child illness. Education about nutrition and counseling provided in ANC during pregnancy can help to reduce the risk of anemia, increase gestational weight gain and improve birth weight [11]. The counseling provided during antenatal care can also promote mothers willingness to register their babies early in under-five clinics, thus possibly promoting good child growth [12].

Weight and length at birth have been reported to be important determinants of infant growth and future nutritional status. Low Birth Weight (LBW) infants have shown difficulties to achieve the standard weight or length at 12 months [13]. Later in life, the prevalence of overweight can be higher in children with LBW i.e. birth weight less than 2500 gram [14].

Exclusive breastfeeding of infants has been shown to give faster growth, regarding both weight and height, during the first 6 months of life compared to weaned and partially breastfed infants [15]. Breastfeeding was associated with a reduced risk of obesity compared to formula feeding in some studies [16]. No effect of prolonged and exclusive breastfeeding on height, adiposity, or blood pressure was observed in a randomized study of Belarusian young school children [17]. Also there was no evidence of causal effects of breastfeeding on body mass index (BMI) and blood pressure in a study aimed to understand the confounding structure of breastfeeding by socio-economic position in the British Avon Longitudinal Study of Parents and Children or the Brazilian Pelotas 1993 cohorts study [18]. Several studies have shown negative associations between the number of infectious disease episodes during infancy mainly pneumonia, diarrhoea and physical growth of the child [19-22].

The main aim for this study is to describe the weight and length growth during a two-year follow-up of children in one urban and one rural cohort in Hanoi, Vietnam and the importance for growth of the three above mentioned immediate factors. The article is also an extension of a previously published article describing growth during the first year of life [23], where differences in birth weight and growth were found between the urban and rural cohorts, between boys and girls and between groups of children with mothers at different educational level and household resources. The article uses information from two earlier studies [23,24] of the mothers’ utilization of antenatal and delivery care as well as breastfeeding practices.

## Methods

### Study sites

All studies were conducted in one rural and one urban area of Hanoi, in northern Vietnam. Dongda is an old, central district of Hanoi. The population is about 352,000 persons. The socio-economic characteristics are typical for the urban areas of big Vietnamese cities. Bavi is a rural district of Hanoi with 250,000 persons, about 60 km from the city center with farming as the main occupation. Two Health and Demographic Surveillance Sites (HDSS) were established to provide information for planning and policy making. The urban HDSS, DodaLab, was started 2007 in three communes with 11,000 households and 38,000 inhabitants [25]. The communes were selected to represent different economic levels. The rural HDSS, FilaBavi, was developed in 1999 using a random sample of 69 clusters including 51,000 persons in 11,000 households [26]. The routine data collection in both sites includes quarterly visits to register vital events and major household surveys every two years to update the socio-economic information about individuals and households.

### Study design and subjects

All mothers with children born alive from 1^st^ March, 2009 to 30^th^ June, 2010, in DodaLab and FilaBavi, were invited to enroll their children in the study. These mothers had taken part in a previous study of antenatal and delivery care [23]. About 99% of the mothers gave consent. The children included were followed from birth to two years of age with respect to weight and length growth, breastfeeding and reported illness. Eight children with congenital or malformation diseases and twelve twins were not invited since their state may influence their birth weight and growth. The interviews made on 88 later out-migrated families and five infants who died during the follow-up period were included. No abnormal characteristics likely to influence growth were observed for these children at birth.

### Data collection

Birth weight information was provided in the first interview when the mothers reported the measurement made at the hospital or commune health centers immediately after birth. For less than 1% of the children birth weight information was not obtained. These children were still used in the postnatal growth analysis.

Totally 1,466 children, 540 in DodaLab and 926 in FilaBavi, were scheduled for monthly measurements of weight and length each month during the first year and every three months during the second year of life. At the same time, mothers were interviewed about breastfeeding and symptoms of illness. The total number of interviews with weight and length measurements was 17,148, 73% of the total 23,456 scheduled. Data about antenatal care and delivery were obtained from the antenatal care study conducted earlier in the two sites [23].

Data about economy and education of mothers were obtained from the household surveys conducted 2009 at the two sites. To describe the household economical level we used the reported yearly household income and the household assets available (according to a specified list) as indicators of economic resources. For the mothers social position we included age, education and occupation. Information about fathers was incomplete since it was not routinely registered [27].

### Concepts, definitions and variables

#### Birth weight

Mothers reported the information they received in hospitals or community health centers immediately after delivery*.* We could not use birth weight information from birth certificate of hospitals or commune health centers. These are kept by the commune administrations in different offices and it was not possible for the field workers to examine all birth certificates. However, we compared the birth weight information from the mothers with the birth certificates in a sample of more than 10% of the infants. The results were found to match very well. The means from the two sources differed about 10 grams. In another sample, information from the hospital records and health centers was used to evaluate the quality of the mothers birth weight information. Again there was no large difference between the different sources of birth weight information.

#### Gestational age

The date of the last menstruation as reported by the mothers was intended to be used for the estimation of the gestational age at birth. This information must however be considered as fairly imprecise since Vietnamese women in general do not remember this date well. It was used nevertheless as a crude proxy for gestational age.

#### Attained weight or length

These are the absolute measurements for a child at any specific child age.

*Stunting, underweight and wasting* in this article are defined as low length-for-age (below mean minus two standard deviations), weight-for-age (below mean minus two standard deviations) and weight-for-length (below mean minus two standard deviations) according to WHO standards [28].

#### Weight and length measurements

##### Child weight

Standardized equipment for measuring the child recommended from Hanoi Medical University was used. A number of commune health center staff members in DodaLab were trained specifically to measure children. In FilaBavi, a number of the permanent interviewers were trained to measure children. The principle of measurement was that the same field worker should assess a child at each visit using the same equipment. Weight was measured to the nearest 10 gram with the child in light clothes using a Vietnamese mechanical infant scale.

##### Child length

Length was measured to the nearest centimeter in horizontal position using a length board. Two person worked together in order to have valid and reliable measurements [27].

*Age* is defined as the date of interview and measurement minus the date of birth.

#### Socio-economic variables

##### Mother’s education

The education of the mother was used as one indicator of the socio-economic situation. Three levels were used: primary school or less, secondary school and higher than secondary school.

##### Household economy

To describe the household economy we investigated different forms of wealth and assets indices and the reported household income. For this study we actually used the number of assets in the following list: bicycle, motorbike, car, telephone, radio, television, video player, sewing machine computer, refrigerator and buffalo.

##### Antenatal care variables

Three variables aimed to describe the use of ANC: (i) The number of antenatal care visits during pregnancy, (ii) ANC reported to contain counseling and advice and (iii) First antenatal visit during first trimester. These variables were found to have the strongest simple correlations to child growth.

##### Breastfeeding variables

Three variables were used to describe breastfeeding during infancy:

*Early initiation of breastfeeding defined as breastfeeding starting during the first hour after birth*[24].

*“Exclusive breastfeeding: The infant receives breast milk, from the breast of the mother or a wet nurse or expressed, with the only additional oral intake of oral rehydration solutions (ORS) or medication including vitamins or minerals”*[29]*.*

*“Any breastfeeding: The infant receives breast milk, from the breast of the mother or a wet nurse or expressed, with or without additional oral foods. This category includes the WHO definitions of exclusive breastfeeding as well as non-exclusive breastfeeding, that is predominant breastfeeding and complementary feeding according to the WHO definitions”*[29]*.*

##### Reported illness symptoms

The indicator reported illness symptoms (fever, cough, diarrhea) at the child level was defined as the number of interviews with one or more reported symptoms divided by number of interviews.

### Statistical analysis

Standard simple and multiple regression models where used for the analysis of birth weight and associations with time and other factors. Mean growth curves were fitted using fractional polynomial (FP) linear regression models [30].

For the analysis of association between attained weight and various factors we used the relative residuals from the predicted curve. The relative residuals were defined as the deviations, positive or negative, of measurements from the FP predicted curve divided by the predicted value.

$$\mathrm{Relative}\;\mathrm{residual}=\left(\mathrm{value}\;\mathrm{observed}–\mathrm{value}\;\mathrm{predicted}\right)/\left(\mathrm{value}\;\mathrm{predicted}\right)$$

Two approaches were used to study associations. This main analysis used the means of residuals for individuals in a collapsed dataset (one record per child). The results were compared to those from repeated measurement analyses using linear mixed regression models applied to all residuals. Results were given as correlations, crude or partial (adjusted). The latter correspond to partial regression coefficients in multiple linear regression but are standardized to have values between minus one and plus one.

All statistical analysis used commands available in the software STATA version 11.

### Ethical considerations

Approval for the project was given by the Scientific and Ethical Committee of Hanoi Medical University, Hanoi Health Bureau and Dong Da district authorities. The data collection in the two sites was approved by the Ministry of Health. The participants were informed about the purpose of the studies and their right to decline participation or withdraw. Consent was obtained from all the study participants. Data was analyzed and presented anonymously. All results have been duly disseminated to communities and authorities.

## Results

Table 1 shows the numbers of children originally involved in the study with some characteristics of the mothers and households. Rather large differences primarily between the urban and the rural site were found. Birth weight means have been reported earlier [27]. Mothers were younger in the rural area and the distributions of the education of the mother differed clearly. The rural mothers had generally lower education than the urban. The households in Dodalab had many more assets than those in FilaBavi. The drop-out rate was dramatically higher in DodaLab than in FilaBavi.

**Table 1:** Characteristics of children, mothers and households in the study

	**Dodalab boys**	**Dodalab girls**	**FilaBavi boys**	**FilaBavi girls**
Number of children enrolled	302	238	515	411
Number of children followed at least one year	170 (56%)	134 (56%)	469 (91%)	361 (88%)
Number of children followed two years	112 (37%)	67 (28%)	443 (86%)	342 (83%)
Mean birth weight of child (grams)	3298	3203	3105	3057
Mother, mean age	28.7	28.3	25.4	25.3
Mother percent primary school	8.6	4.9	54.8	54.6
Mother percent secondary school	33.2	28.0	27.8	28.6
Mother percent higher education	58.2	67.1	17.4	16.8
Number of household assets (means)	9.4	9.1	4.7	4.8

### Attained weight and length of children during first two years of life

**Figure 2:**
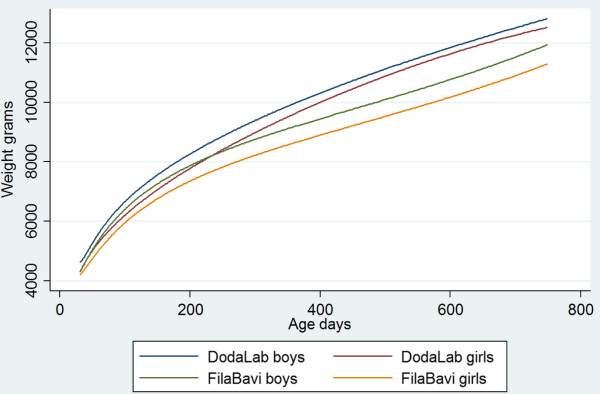
Estimated weight (grams) as functions of age by site and child sex.

**Figure 3:**
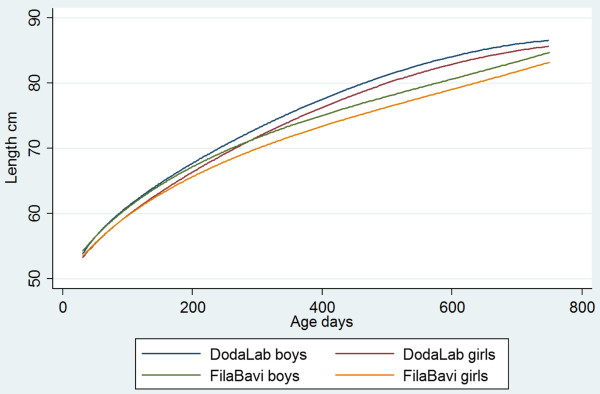
Estimated length (cm) as functions of age by site and sex.

Figures 2 and 3 show the fitted growth curves from 2 months age to two years. The urban weight curve exceeds the rural with about 5% at one year of age, similarly for boys and girls. The gap decreases during the second year. The curves differ statistically significantly between the urban and rural children as well as between boys and girls. The curves showing the mean attained weight according to WHO growth standards [31] fall between the urban and rural curves.

Table 2 shows the percentages for measurements indicating stunting by some factors of interest and four age groups. In general stunting measurements were most frequent in boys and children having mothers with low education and low level of household assets. There were also differences between the urban and rural areas.

**Table 2:** Percentages of length measurements indicating stunting according to WHO definition and the WHO 2006 growth standard

	**First half year**	**Second half year**	**Third half year**	**Fourth half year**	**Total**
Boys	12.0	12.7	14.2	21.4	14.1
Girls	8.2	8.9	11.4	19.8	10.7
Urban area	11.3	8.8	2.6	6.8	8.8
Rural area	9.9	12.2	18.4	26.4	14.6
Low education	9.2	13.2	19.6	28.5	15.2
Middle education	11.5	11.4	12.4	20.1	13.0
High education	10.3	7.9	6.7	9.5	8.8
Low assets	10.5	13.4	18.4	26.0	14.5
Middle assets	11.0	10.8	13.8	22.0	14.9
High assets	8.0	8.6	6.5	11.4	9.4
Total	10.3	11.0	13.0	20.7	12.4

### Growth and antenatal care

The strongest positive correlations between weight and length and the ANC variables were with the number of ANC visits and a positive answer to the question if the mother was given advice and counseling which was reported by 44% of all women (Table 3). The partial correlation coefficients, adjusted for site sex, education and assets, differ markedly from the overall. Only the partial correlation between length and number of ANC visits remained statistically significant. A further breakdown is given for weight in Table 4.

**Table 3:** Simple (unadjusted) correlation coefficients and partial (adjusted) correlation coefficients between weight and length residuals and selected explanatory variables

	**Attained weight**	**Attained weight adjusted**	**Attained length**	**Attained length adjusted**
Number of ANC visits	.1979	.0379	.1790	.0762
Advice during ANC	.1859	-.0063	.0923	-.0392
Early initiation of breast-feeding	.0687	.0790	.0546	.0359
Duration of exclusive breastfeeding	.1211	.0404	-.0051	-.0200
Reported illness symptoms	-.3076	-.1769	-.1770	-.0808

“Adjustment for site, sex mother education and household assets”.

**Table 4:** Correlations between weight residuals and selected variables, adjusted for mother education and assets by site and sex

	**Urban boys**	**Urban girls**	**Rural boys**	**Rural girls**
Number of ANC visits	.0340	.0003	.0942	.0378
Early initiation of breast-feeding	.0224	.0575	.0914	.1653
Reported illness symptoms	-.0803	-.0674	-.2165	-.1788

### Growth and breastfeeding

Early initiation of breastfeeding and the duration of exclusive breastfeeding were positively correlated to weight growth. For length the latter correlation was small and insignificant (Table 3). After adjustment for site, child sex, mother education and household assets only the correlation between weight growth and early initiation of breastfeeding was statistically significant. The detailed account in Table 4 suggests that early initiation of breastfeeding is more strongly correlated to weight growth in the rural area.

### Growth and reported illness symptoms

Increasing numbers of interviews with reported symptoms was statistically significantly associated with reduced weight growth (Table 3). Illness was much more commonly reported in the rural area where the risk was over 0.40 compared to about 0.20 in the urban. The negative correlations with weight growth were considerably stronger in the rural area (Table 3).

## Discussion

One finding of this paper is that the differences in growth between children in the studied Vietnamese urban and rural areas previously reported during the first year of life [27], remain at two years of age. In addition, we found a positive association between weight growth and early initiation of breastfeeding and a negative association with reported illness symptoms.

Much evidence supports that breastfeeding provides good nutrition for children as it reduces the severity of e.g. respiratory and gastrointestinal infections in children [32-34]. Children with exclusive breastfeeding have been seen to grow better [15] and breastfeeding can be associated with reduced risk of obesity later in life compared with formula fed infants [16]. One suggested reason is that breastfeeding protects through activity of specific components of breast milk such as hormones involved in appetite and energy balance [35].

Poor nutrition has been seen as the most important risk for poor growth [36] and differences in nutrition between urban and rural areas could be the main reason for the observed differences in this study. During the last decades, the Vietnamese dietary intake has improved in both quality and quantitive through consumption of food such as fish, meat, fat oils, etc. [37]. However, differences in food consumption between urban and rural areas in Vietnam have been reported [38,39]. Deficiency of iron, calcium, phosphorus, potassium, magnesium, beta- carotene, vitamin A and vitamin C has been found in Vietnamese rural girls 7–9 years old in spite of adequate consumption of all these elements except low carotene [38]. The nutritional status of under five children is proposed as a sensitive indicator of household economic condition and parent’s education [40]. Differences in nutrition between the urban and rural areas could be a strong reason for the observed growth differences.

According to WHO and UNICEF, the prevalence of stunting among children under five in Asia was 27% in 2011 [28]. In Vietnam, one third of children under five were stunted [21]. In the present study, the percentage of measurements indicating stunting two years after birth was over 20% indicating that the prevalence of stunted children is quite high.

The Vietnamese government has noted stunting as a public health problem. A plan to reduce the incidence of stunting to 23% by 2020 and underweight to 12.5% by the same year in children under five was launched in 2012 [41]. A contributing factor for the high stunting in boys may be that boys are less breastfed than girls [24]. The reason for this can be that mothers consider boys to be more important than girls and at the same time think that formula feeding is better than breastfeeding [24].

We observed some statistically significant simple (unadjusted) correlations between growth and ANC use. In, regression models where both underlying and immediate variables are included though, the education and assets variables turn out to be more important than the ANC indicators. The partial correlations between growth and ANC use, adjusting for education level of the mother and the household resources, are small and not statistically significant. The simple explanation can be that the ANC variables are themselves associated with education and economy. Socially and economically resourceful mothers possibly use, and benefit, more from ANC than others.

Child illness stands out among the studied immediate factors. A fairly strong association between growth of children and reported illness symptoms was found, particularly in the rural area.

Symptoms of illness were most commonly reported in the rural area. The risk for illness reporting at a particular visit was 0.40 compared to about 0.20 in the urban. The high incidence of illness could be important to explain the slow growth of rural infants. The possible negative influence on weight growth was also stronger in the rural area.

The most common causes of illness in children under five and especially during infancy are diarrhea and acute respiratory infection. This has been observed in several studies [42-44]. Diarrhoea was concluded to drastically reduce the growth velocity in weight and length e.g. in a Brazilian study [20] where diarrhoea during the first six months increased the risk of low BMI and weight for length later. Diarrhea after six months of age increased the risk for low weight for age and stunting in a Vietnamese study [21]. Acute respiratory infection has also been seen to be significantly associated with incremental weight loss of infants e.g. in Indonesia [22].

A possible intervention may be that the Vietnamese Ministry of Health work to enhance the quality of the Integrated Management of Childhood Illness program aimed to help lay community health workers assess and treat sick children. Improvements of health staff skills as well as the health system itself seem also to be needed particularly in the rural area.

Early initiation of breastfeeding, within the first hour of life, can be a positive factor for growth and has been claimed to protect newborn from acquiring infections [45]. The main reason why breastfeeding protects babies from infectious diseases is that it modulates the early exposure of neonate’s intestinal mucosa to microbes and limits bacterial translocation through the gut mucosa [46]. Being more common in the urban than in the rural area [24], early initiation of breastfeeding can be another reason for the different growth of infants in rural and urban area.

Exclusively breastfed infants have been seen to grow faster during the first 6 months of life compared to groups of weaned and partially breastfed children [15,47]. Exclusive breastfeeding can decrease the number of diarrhea and acute respiratory infection episodes [48]. The duration of exclusive breastfeeding though, does not relate to growth in the present study. A reason may be that the duration is short in both areas; less than two months for most children.

Decisions taken by mothers about use of ANC, breastfeeding, nutrition and child health care utilization are related to the educational level of mothers and the household resources. Likewise, the risks for illness are associated with education and economy. In the present study about 19% of the variation in weight growth and 12% for length growth are explained by the variation in education of the mother and household wealth. Adding the ANC indicators, early initiation of breastfeeding and illness symptoms as independent variables in the regression model increased the determination coefficient by about two percent units. However, more than 80% of the total growth variation is left unexplained.

The associations between growth and the immediate factors in the conceptual framework for this study are to large extents reflections of the associations between growth and the underlying socio-economic factors. Thus these turn out to be the most important to explain growth variation. Interventions aimed at improved provision and use of antenatal care, promoting good breastfeeding practices and preventing child morbidity will have their effects. These risk to be limited as long as the underlying social and economic conditions are not strong and equitable. In the end the basic factors with its political, social, economic, cultural and other contexts will determine the conditions for child growth.

A child with a complete weight and length measurement set has been measured 12 times during the first year of life and four times during the second. As can be seen in Table 1, complete sets were not received in 34.2% of the included children. The most common reasons for dropout was that the visit to the household could not take place for practical reasons or the mother declined to cooperate.

The dropout rates were clearly different between the urban and the rural area and to some extent between boys and girls in the rural area. The first mentioned difference could be expected since many mothers in the urban area work outside the household and visits could be more difficult to arrange.

The dropouts cannot be expected to be random but systematic and possibly creating bias. To investigate we compared the growth curves fitted using all available observations with other curves using only the data from children with complete sets. The former curves came out systematically lower than the latter but the differences were small, 30 to 50 gram after two years of age, largest in DodaLab. Another approach used to investigate possible bias was to correlate the means of relative residuals for the first half-year to the number of visits. Very weak positive correlations were found. Both approaches thus indicate that the risk to dropout is higher for children with slower growth. Correlations between birth weight and number of measurements however, did not support that conclusion.

The study is to a large extent dependent on information reported by the mothers, e.g. birth weight. . Possible sources of errors are both how the measured birth weight was reported to the mother and the recall of mothers. It could be suspected that the hospital or health center staff tends to report a higher weight to please the mother. The proportion of low birth weight newborn (birth weight below 2,500 gram) is lower than expected. On the other hand there is no heaping e.g. at 2,500 gram in the birth weight distribution. The precision of birth weight reporting is 100 gram. Systematically and incorrectly rounding upwards would create a bias of that size. Recall biases are likely to be small as it is considered important for a mother to remember the birth weight of a child in the Vietnamese tradition. Another important piece of information provided by the mothers was the date of the last menstruation before pregnancy. This is needed to calculate the gestational age at birth. The adequacy of this information turned out to be somewhat problematic. The information is missing for quite many women and it seems that by using the available information we underestimate the gestational age. Too many women appear to be classified as giving birth prematurely. However, using or not using the gestational age as an explanatory variable for growth does influence the results only marginally.

Household assets have been used as an indicator of household economical resources. The available alternatives could be the reported household incomes or reported expenditures. A third alternative could be the “Wealth index” which is a wider combination of housing characteristics and assets [49,50]. All these indicators have been tried in the present analysis, one by one and in combinations, although they are strongly correlated. The asset index was concluded to have the strongest correlation to growth. We also tried to use both assets and household income in the same model. Then the correlations with assets variable come out statistically significant whereas correlations with income are smaller and non-significant.

## Conclusion

Globalization and urbanization means obvious risks for increasing social and economic gaps between urban and rural areas. The predictors of growth discussed studied in this article, antenatal care, breastfeeding and reported illness, are associated to social and economic conditions as underlying factors. In order to improve and maintain good conditions for child growth it is important to strengthen the education of mothers and the household resources, particularly in the rural areas. The high prevalence of stunting observed underscores this. In addition, improvement of the quality of programs for antenatal care, postnatal care, breastfeeding and integrated management of childhood illness are needed in Vietnam.

## Competing interests

The authors declare that our findings have not been influenced by our personal or financial relationship with other person or other organization.

## Authors’ contributions

HNT led and supervised the fieldwork and data management. She also drafted and completed this paper. BE assisted in the research design as well as in the statistical analyses, interpretation of results and revision of the manuscript. HA, LNT, MP, TTK and GB were involved in the design of the study, supervised the study and revised the manuscript. All authors have read and approved the final manuscript.

## Pre-publication history

The pre-publication history for this paper can be accessed here:

http://www.biomedcentral.com/1471-2431/13/149/prepub
